# Salternamide A Suppresses Hypoxia-Induced Accumulation of HIF-1α and Induces Apoptosis in Human Colorectal Cancer Cells

**DOI:** 10.3390/md13116962

**Published:** 2015-11-19

**Authors:** Duc-Hiep Bach, Seong-Hwan Kim, Ji-Young Hong, Hyen Joo Park, Dong-Chan Oh, Sang Kook Lee

**Affiliations:** College of Pharmacy, Seoul National University, Seoul 151-742, Korea; E-Mails: bdhiep90@snu.ac.kr (D.-H.B.); yanberk@snu.ac.kr (S.-H.K.); jyhong7876@daum.net (J.-Y.H.); phj00@snu.ac.kr (H.J.P.); dongchanoh@snu.ac.kr (D.-C.O.)

**Keywords:** salternamide A (SA), HIF-1α, PI3K/Akt/mTOR, p42/p44 MAPK, STAT3, cell death

## Abstract

Hypoxia inducible factor-1α (HIF-1α) is an essential regulator of the cellular response to low oxygen concentrations, activating a broad range of genes that provide adaptive responses to oxygen deprivation. HIF-1α is overexpressed in various cancers and therefore represents a considerable chemotherapeutic target. Salternamide A (SA), a novel small molecule that is isolated from a halophilic *Streptomyces* sp., is a potent cytotoxic agent against a variety of human cancer cell lines. However, the mechanisms by which SA inhibits tumor growth remain to be elucidated. In the present study, we demonstrate that SA efficiently inhibits the hypoxia-induced accumulation of HIF-1α in a time- and concentration-dependent manner in various human cancer cells. In addition, SA suppresses the upstream signaling of HIF-1α, such as PI3K/Akt/mTOR, p42/p44 MAPK, and STAT3 signaling under hypoxic conditions. Furthermore, we found that SA induces cell death by stimulating G2/M cell cycle arrest and apoptosis in human colorectal cancer cells. Taken together, SA was identified as a novel small molecule HIF-1α inhibitor from marine natural products and is potentially a leading candidate in the development of anticancer agents.

## 1. Introduction

The transcription factor hypoxia-inducible factor-1 (HIF-1) plays a pivotal role in regulating the initiation of genes that are involved in decisive aspects of cancer biology, such as angiogenesis, cell survival, differentiation, invasion, tumor progression, and glucose metabolism [1,2,3,4,5,6]. HIF-1 is a heterodimer that consists of HIF-1α and HIF-1β subunits. In addition, HIF-1 activity in tumors depends on the availability of the HIF-1α subunit and the levels of HIF-1α expression under hypoxic conditions [4]. Indeed, HIF-1α is overexpressed in a variety of human cancers compared to normal tissues [7,8]. Consequently, HIF-1α is an appealing intracellular target for treating a wide range of hypoxia-related pathologies by targeted cancer therapy [9].

The overexpression of HIF-1α is due to the fundamental interaction between various metabolic pathways and factors that lead to particular genetic alterations and extracellular stimuli, such as hypoxia that impact both protein degradation and synthesis [10]. Two main signaling pathways are involved in the regulation of HIF-1α function and protein levels: the phosphatidylinositol 3-kinase (PI3K) and the mitogen-activated protein kinase (MAPK) pathways [4]. Signaling from the PI3K pathway enhances HIF-1α synthesis through the mammalian target of the rapamycin protein complex (mTOR; a kinase that functions downstream of PI3K and Akt), likely by heightening HIF-1α translation. The p42/p44 MAPK pathway may induce the transactivation function of HIF-1α through the direct phosphorylation of HIF-1α [11] or by upregulating its cofactor p300 [12]. Additionally, recent studies have reported that the signal transducer and activator of transcription-3 (STAT3) is activated in response to hypoxia, a common feature of various solid tumors [13,14]. Activated STAT3 also mediates the upregulation of HIF-1α by enhancing its transcriptional activity [15].

Salternamide A (SA), a novel small molecule marine agent, was recently isolated by our group from a halophilic *Streptomyces* sp. possessing anti-proliferative activity against various cancer cells [16]. SA was identified as the first secondary metabolite from a saltern-derived actinomycetes microorganism and the first chlorinated member of the manumycin family. However, there has been no report further evaluating its anticancer activity and mechanisms of action in human colon cancer cells.

In the present study, we attempted to investigate the mechanism by which SA suppresses HIF-1α protein accumulation and induces cell death in HCT116 human colon cancer cells.

## 2. Results and Discussion

### 2.1. Salternamide A Suppresses Hypoxia-Induced HIF-1α Protein Accumulation in Various Cancer Cells

**Figure 1:**
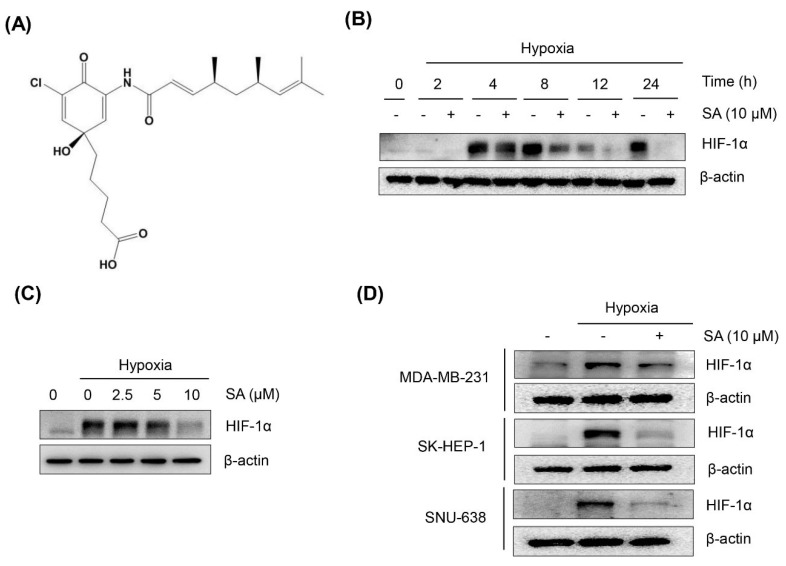
Effect of SA on hypoxia-induced HIF-1α protein accumulation in various cancer cells. (**A**) Chemical structure of SA; (**B**) HCT116 cells were treated at the indicated time points under normoxic or hypoxic conditions (CoCl_2_ treatment) in the presence or absence of SA (10 μM); (**C**) HCT116 cells were treated for 8 h under normoxic or hypoxic conditions in the presence or absence of increasing SA concentrations; (**D**) MDA-MB-231, SK-HEP-1, and SNU-638 cells were treated with 10 μM SA for 8 h under normoxic or hypoxic conditions. Immunoblotting analysis was performed to determine HIF-1α and β-actin protein levels.

To investigate whether SA (Figure 1A) affects HIF-1α induced by hypoxia, HCT116 cells were exposed to normoxic or hypoxic (CoCl_2_ treatment) conditions for 2, 4, 8, 12, or 24 h in the presence of 10 μM SA. As shown in Figure 1B, HIF-1α expression was significantly induced by hypoxia-mimetic CoCl_2_ treatment, starting from as early as 4 h. However, SA effectively suppressed hypoxia-induced HIF-1α protein expression at 8 h along with marked suppression at 12 and 24 h (Figure 1B). In addition, when treated with SA for 8 h under hypoxic conditions, SA suppressed the accumulation of hypoxia-induced HIF-1α protein in a concentration-dependent manner (Figure 1C).

To further examine whether the suppressive effect of SA on HIF-1α expression is applicable to a variety of cancer cell lines with different genetic backgrounds (wild-type or mutated p53), given that HIF-1α is destabilized by p53 [17] in different organs, SK-HEP-1 (liver), SNU-638 (gastric), and MDA-MB-231 (breast) cancer cells were treated with 10 μM SA for 8 h. SA effectively suppressed the expression of HIF-1α in the tested cancer cells, similar to the results shown in HCT116 cells (Figure 1D). These findings suggest that SA suppresses HIF-1α expression in various cancer cell types by blocking HIF-1α protein accumulation in response to hypoxic conditions.

### 2.2. Suppression of HIF-1α Accumulation by Salternamide A in HCT116 Cells Is Independent of Proteasomal Degradation

**Figure 2:**
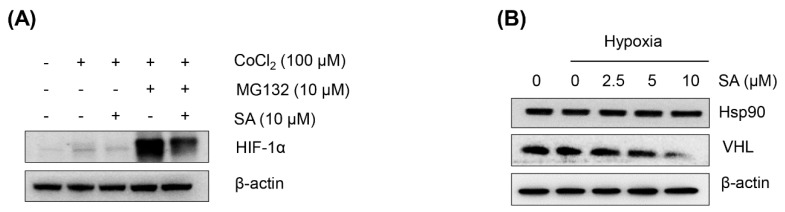
Effect of SA on the degradation of HIF-1α. (**A**) HCT116 cells were treated with a proteasome inhibitor (10 μM MG132) and 10 μM SA under normoxic or hypoxic conditions before immunoblotting; (**B**) for VHL and Hsp90 immunoblotting, HCT116 cells were treated with SA and cultured for 8 h under normoxic or hypoxic conditions, respectively.

In general, the accumulation of HIF-1α depends on the balance between its degradation and synthesis (translation) [18]. To determine whether SA is able to suppress HIF-1α protein accumulation by promoting its degradation, the cells were pretreated with the proteasome inhibitor MG132, followed by SA treatment in HCT116 cells. As shown in Figure 2A, pretreatment with MG132 resulted in the accumulation of HIF-1α, but SA efficiently abrogated the accumulation of HIF-1α despite proteasome suppression, indicating that SA decreases HIF-1α protein accumulation through a pathway independent of proteasomal degradation.

The von Hippel-Lindau (VHL) tumor suppressor protein recruits an E3-ubiquitin ligase that targets HIF-1α for proteasomal degradation [4]. In addition, heat-shock protein 90 (Hsp90) binds to HIF-1α and promotes its stability [19]. To determine whether the suppression of HIF-1α protein expression by SA is associated with these adaptor proteins, Western blot analysis was performed under hypoxic conditions with the treatment of SA in HCT116 cells. As a result, SA did not significantly enhance the VHL or abrogate Hsp90 expression in the HCT116 cells (Figure 2B). These data suggest that the suppression of HIF-1α accumulation by SA under hypoxic conditions might not be associated with the enhancement of the degradation of HIF-1α under these conditions. Further study revealed that SA did not affect HIF-1α gene transcription or HIF-1α mRNA stability (data not shown). Overall, the suppressive effect of SA on the accumulation of HIF-1α protein expression under hypoxic conditions might be due in part to the downregulation of the translation of HIF-1α mRNA. These translational regulations should be further clarified in detail.

### 2.3. Salternamide A Suppresses the Hypoxia-Induced Accumulation of HIF-1α via the Regulation of Signal Transduction Pathways

**Figure 3:**
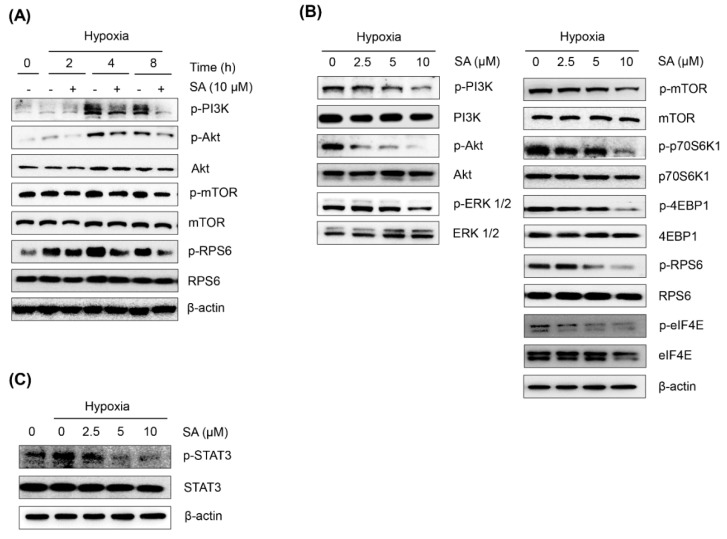
Effect of SA on hypoxia-response protein expressions. (**A**) HCT116 cells were treated at the indicated time points under normoxic or hypoxic conditions in the presence or absence of SA (10 μM) before immunoblotting. (**B** and **C**) HCT116 cells were treated for 8 h under normoxic or hypoxic conditions in the presence or absence of increasing SA concentrations.

Recent studies have reported that the PI3K/Akt/mTOR and p42/p44 MAPK pathways are associated with the regulation of HIF-1α protein synthesis at the translational level [10,20]. The p42/p44 MAPK also enhances the transcriptional activity of HIF-1α [11]. To address the potential involvement of these pathways in the SA-mediated suppression of HIF-1α accumulation, the expression of the proteins in the signal transduction pathway was determined by Western blot analysis. As shown in Figure 3A, activated (phosphorylated form) PI3K, Akt, mTOR, and RPS6 expression under hypoxic conditions was downregulated by the treatment of SA (10 μM) in a time-dependent manner. A subsequent study also revealed that SA effectively suppressed the expressions of these signaling proteins in a concentration-dependent manner (Figure 3B). In addition, SA also downregulated the activation of p70S6K1 (Thr^389^), 4E-BP1 (Thr^37/46^), eIF4E (Ser^209^), and RPS6 (Ser^235/236^), which are downstream target molecules of mTOR complex 1 (mTORC1) signaling pathways. Therefore, these data suggest that the suppression of HIF-1α protein expression by SA might be partly associated with the downregulation of mTORC1 signaling pathways under hypoxic conditions.

Recent reports have also revealed that the transcription factor STAT3 is involved in the transcriptional regulation of HIF-1α and that the activation of STAT3 activity is enhanced by hypoxia [21,22]. In the present study, we also found that hypoxia enhanced STAT3 activation (phosphorylated at the Tyr^705^ residue), and this effect was abrogated by the treatment of SA in a concentration-dependent manner (Figure 3C).

Overall, these findings suggest that the suppression of the PI3K/Akt/mTOR, p42/p44 MAPK, and STAT3 signaling pathways by SA might be associated, in part, with the downregulation of HIF-1α protein synthesis.

### 2.4. Salternamide A Induces Apoptotic Cell Death in Human Colorectal Cancer Cells

**Figure 4:**
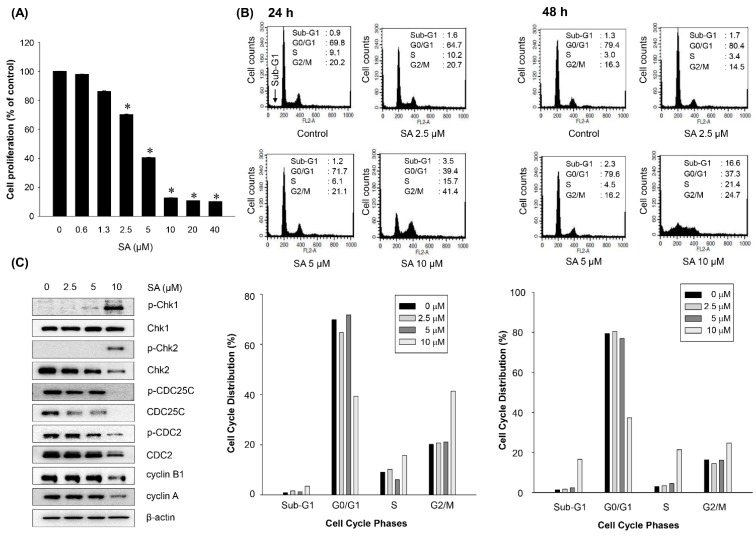
Effect of SA on cell cycle distribution in HCT116 human colorectal cancer cells. (**A**) Anti-proliferative effect of SA on HCT116 cells. HCT116 cells were treated with various concentrations of SA for 72 h, and cell proliferation was determined with the SRB assay. The data represent the mean percentage ± SD compared to the DMSO-treated control group. Each experiment was performed in triplicate (*n =* 3). * *p* < 0.05 compared with the control group. (**B**) HCT116 cells were treated with SA for 24 or 48 h. The cell cycle distribution was analyzed by flow cytometry. (**C**) Effects of SA on the expression of cell cycle-related proteins in HCT116 colorectal cancer cells. HCT116 cells (2 × 10^5^ cells/mL) were treated with SA for 24 h. Subsequently, the protein expression levels of cell cycle-related proteins were analyzed by Western blotting.

A previous study revealed the anti-proliferative activity of SA in a panel of cancer cell lines [16]. To further elucidate the mechanism of action of the anti-proliferative activity of SA, the effect of SA on the regulation of the cell cycle was determined in HCT116 cells. When treated with SA for 72 h, SA primarily inhibited the growth of HCT116 cells in a concentration-dependent manner (Figure 4A). SA (10 μM) increased the cell population in the sub-G1 phase at 48 h (Figure 4B, right panel). To further elucidate whether cell cycle arrest is associated with the regulation of cell cycle checkpoint proteins, the expression of G2/M cell cycle regulatory proteins was analyzed by Western blotting. As shown in Figure 4C, the expression of p-CDC25C (Ser^216^), CDC25C, p-CDC2 (Thr^161^), CDC2, cyclin B1, and cyclin A were significantly suppressed, but the levels of p-chk1 (Ser^345^) and p-chk2 (Thr^168^) were upregulated by the treatment of SA. These findings suggest that the anti-proliferative activity of SA is associated, in part, with the induction of the G2/M phase cell cycle arrest by modulating cell cycle regulators in HCT116 cells.

**Figure 5:**
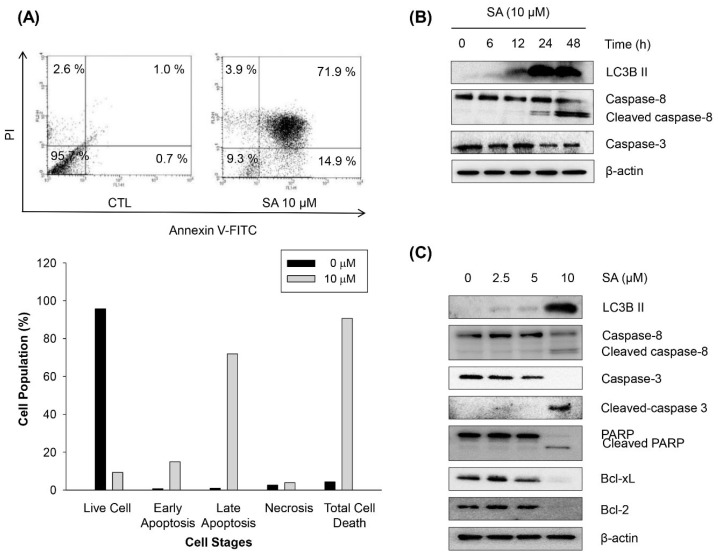
Effects of SA on apoptosis and autophagy in HCT116 human colorectal cancer cells. (**A**) HCT116 cells (1 × 10^5^ cells/mL) were treated with SA for 48 h. Following treatment, HCT116 cells were harvested and stained with Annexin V-FITC and PI and analyzed using flow cytometry as described in the experimental section. (**B**) HCT116 cells were treated at the indicated time points in the presence or absence of SA (10 μM) prior to Western blotting. (**C**) Effects of SA on the expression of apoptosis- and autophagy-related proteins in HCT116 colorectal cancer cells. HCT116 cells (2 × 10^5^ cells/mL) were treated with SA for 48 h; subsequently, the protein expression levels of apoptosis- and autophagy-related proteins were analyzed by Western blotting.

To further confirm whether the induction of the sub-G1 peak by SA (10 μM) at 48 h is related to apoptotic cell death, the cells were treated with SA (10 μM) for 48 h, and the quantification of Annexin-V/PI staining, a marker for apoptosis, was determined by flow cytometry. The number of cells that were positive for the double staining of Annexin-V/PI was significantly increased by SA treatment, suggesting that SA is able to induce apoptotic cell death in cancer cells (Figure 5A). To further unveil the mechanism of SA-induced apoptosis in HCT116 cells, the expression of apoptosis-associated proteins was analyzed by Western blotting. As shown in Figure 5B,C, the expression of cleaved caspase-8, cleaved caspase-3, and cleaved PARP was upregulated, but the expression of pro-caspase-8, caspase-3, pro-PARP, Bcl-2 and Bcl-xL was downregulated in a time- and concentration-dependent manner. In addition, LC3-II is an autophagy-specific marker, and LC3-II formation (LC3 lipidation) is also a key step in autophagy-associated cell death [23]. To evaluate whether the cytotoxic activity of SA is related, in part, to the induction of autophagy, the expression level of LC3-II was determined by 48 h of SA (10 μM) treatment. SA also significantly induced the expression of LC3-II in a time- and concentration-dependent manner. Taken together, these results suggest that the anti-proliferative activity of SA might be due, in part, to the G2/M phase cell cycle arrest and the induction of apoptosis and autophagic cell death in HCT116 cells.

### 2.5. Discussion

Many efforts have been designed to identify and develop new small molecule HIF-1α inhibitors. Natural products have played an important role in drug discovery and development by providing novel chemical entities. In the present study, we found that salternamide A (SA) is a novel HIF-1α inhibitor derived from natural sources. SA is one of the salternamides that we recently isolated from a halophilic *Streptomyces* sp. of saltern-derived actinomycetes [16]. In terms of chemical characteristics, SA is novel and is the first chlorinated member of the manumycin class. Initially, SA exhibited the most potent cytotoxicity among the isolated salternamides against a panel of cancer cell lines [16]. However, the precise mechanism of action of the anti-proliferative activity of SA is not fully understood. Based on the unique chemical structure and potent growth inhibition of cancer cells, we attempted to elucidate its mechanism of action in cancer cells. From the initial analysis of chemical structures of salternamides with the amide-side chain, we assumed that HIF-1α should be a candidate target molecule. As a result, SA suppressed the accumulation of the HIF-1α protein under hypoxic conditions in human colorectal HCT116 cancer cells.

VHL is the substrate recognition component of an E3 ubiquitin ligase complex that targets HIF-1α and HIF-2α for ubiquitin-mediated degradation under normoxic conditions. The interaction of pVHL (the protein transcript of the VHL gene) with HIF-1α is one of several O_2_-dependent events thought to regulate HIF activity [24]. Inactivation of the VHL gene is correlated to the development of highly vascularized tumors. pVHL directs the polyubiquitylation of HIF-1α for ubiquitin-mediated degradation in an oxygen-dependent manner. Therefore, loss of VHL is sufficient not only to stabilize HIF-1α subunits under normoxia, but also to fully activate HIF-mediated responses [24]. Maranchie *et al*. [25], however, reported that loss of the tumor suppressor VHL did not enhance the growth of subcutaneous tumors derived from primary cells. In the present study, the level of VHL was decreased by SA at 10 μM, suggesting that the inhibition of HIF-1α accumulation by SA under hypoxic conditions might not be correlated to the increasing of the degradation of HIF-1α. However, the detailed mechanisms of this action need to be further clarified. Further study revealed that the suppressive effect of SA on the accumulation of HIF-1α might be partly due to the inhibition of the translation of HIF-1α, without affecting the expression level of HIF-1α mRNA or the degradation of the HIF-1α protein.

The expression of HIF-1α is closely regulated by both its protein synthesis and degradation. The translation of HIF-1α is promoted by the axis of mTOR signaling through the activation of p70S6K, 4E-BP1, and eIF4E [26]. The PI3K/Akt/mTOR and p42/p44 MAPK pathways also activate translational regulatory proteins, including 4E-BP1, eIF4E, and p60S6K [4]. SA modulates the mTOR signaling pathways and the axis of PI3K/Akt/MAPK-mediated downstream signaling, thus leading to the suppression HIF-1α translation under hypoxic conditions in cancer cells.

Recent studies have also reported that STAT3 is activated in response to hypoxia in various cancers [13,14], and STAT3 signaling is required for HIF-1α protein accumulation in the activation of endogenous HIF-1α target genes [27,28]. In the present study, SA suppressed the activation of STAT3, which led to the downregulation of HIF-1α protein expression under hypoxic conditions. These findings also suggest that the regulation of STAT3 by SA is partly involved in the suppression of HIF-1α accumulation under hypoxic conditions in cancer cells.

Taken together, although further detailed study is needed to elucidate how the translation of HIF-1α is regulated, SA is considered a novel inhibitor of HIF-1α protein synthesis under hypoxic conditions in cancer cells.

In addition, the anti-proliferative activity of SA was associated with G2/M cell cycle arrest and subsequent apoptotic cell death in HCT116 cancer cells. G2/M phase arrest was highly correlated with the downregulation of CDC25C and CDC2 checkpoint protein expression [27]. The progression from G2 to M phase is regulated by a number of cyclin family members, such as cyclin B1 and CDC2. Cyclin B1, together with cyclin A, promotes the G2/M transition [29,30]. Meanwhile, CDC2 is crucial for the G1/S and G2/M phase transitions of the eukaryotic cell cycle. The phosphatase activity of CDC25C is also implicated in the regulation of the progression of G2/M phase [31]. Chk1 (Checkpoint Kinase 1) and Chk2 (Checkpoint Kinase 2) are multi-functional protein kinases that coordinate the response to specific types of DNA damage [32]. As a result, we found that SA-mediated G2/M phase cell cycle arrest was associated with the regulation of CDC25C and CDC2 and its related checkpoint kinases Chk1/2.

Apoptosis is one of the main types of programmed cell death, and many anti-cancer agents induce apoptosis against cancer cells to achieve therapeutic efficacy [33,34]. We also found that longer and higher concentrations of SA exposure are able to induce apoptotic cell death in the HCT116 cells. Apoptotic cell death by SA was confirmed by significant increases in the proteolytic cleavage of caspase-8, -3 and PARP, and the downregulation of the antiapoptotic Bcl-2 and Bcl-xL proteins.

Autophagy plays a complex role in cancer development, with a tumor-progressive or a tumor-suppressive effect [35]. Apoptosis and autophagy may be interconnected, either antagonistically or cooperatively, in response to various anti-cancer therapeutics in different cancer cells [36]. Herein, we observed that the formation of LC3-II, a marker of autophagy, was also induced by SA, suggesting that autophagy-mediated cell death is also involved in the anti-proliferative activity of SA in HCT116 cancer cells.

## 3. Materials and Methods

### 3.1. Materials

Salternamide A (SA, Figure 1A) was dissolved in 100% DMSO and stored at −20 °C for subsequent analysis. Cobalt (II) chloride (CoCl_2_) and MG132 were purchased from Sigma-Aldrich (St. Louis, MO, USA). Antibodies for HIF-1α, Akt, phospho-Akt (Thr^308^), PI3K, phospho-PI3K (Tyr^458/199^), RPS6, phospho-RPS6 (Ser^235/236^), p70S6K1, phospho-p70S6K1 (Thr^389^), phospho-STAT3 (Tyr^705^), mTOR, phospho-mTOR (Ser^2448^), 4E-BP1, phospho-4E-BP1 (Thr^37/46^), eIF4E, phospho-eIF4E (Ser^209^), phospho-CDC2 (Thr^161^), CDC25C, phospho-CDC25C (Ser^216^), Chk1, phospho-Chk1 (Ser^345^), phospho-Chk2 (Thr^168^), caspase-3, caspase-8, caspase-9, cleaved caspase-3, cleaved caspase-8, and LC3B were purchased from Cell Signaling Technology (Danvers, MA, USA). Antibodies for ERK 1/2, phospho-ERK 1/2 (Thr^202^/Tyr^204^), STAT3, CDC2, cyclin B1, cyclin A, Bcl-2, and Bcl-xL were purchased from Santa Cruz Biotechnology (Santa Cruz, CA, USA). Antibodies for VHL, PARP, cleaved PARP, and Bim were purchased from BD Pharmingen™ (BD Biosciences, San Jose, CA, USA). Hsp90 antibody was purchased from Stressgen Bioreagents (Ann Arbor, MI, USA).

### 3.2. Cell Culture

Human colorectal cancer cells (HCT116), gastric cancer cells (SNU638), breast cancer cells (MDA-MB-231), and liver cancer cells (SK-HEP-1) were purchased from American Type Culture Collection (Manassas, VA, USA). HCT116 and SNU638 cells were maintained in RPMI1640 medium, while MDA-MB-231 and SK-HEP-1 cells were cultured in DMEM medium that was supplemented with 10% heat-inactivated fetal bovine serum (FBS), 100 units/mL penicillin, 100 μg/mL streptomycin, and 250 ng/mL amphotericin B, respectively. Cells were maintained at 37 °C in a humidified atmosphere with 5% CO_2_.

### 3.3. Sulforhodamine B Assay

HCT116 cells (2 × 10^4^ cells/mL) were seeded in 96-well plates with various concentrations of SA and incubated at 37 °C in a humidified atmosphere with 5% CO_2_. After incubation, the cells were fixed with a 50% trichloroacetic acid (TCA) solution for 1 h, and cellular proteins were stained with 0.4% sulforhodamine B (SRB) in 1% acetic acid. The stained cells were dissolved in 10 mM Tris buffer (pH 10.0). The effect of SA on cell proliferation was calculated as a percentage relative to a solvent-treated control, and the IC_50_ values were evaluated using nonlinear regression analysis (percent survival *versus* concentration).

### 3.4. Flow Cytometry for Cell Cycle and Apoptosis Analysis

HCT116 cells (2 × 10^5^ cells/mL) were plated in a 36-mm culture dish and incubated for 24 h. Fresh medium containing the indicated concentration of SA was added to culture dishes. Following a 24 or 48 h incubation, the cells were harvested (via trypsinization and centrifugation), rinsed twice with pre-cooled phosphate buffered saline (PBS), and prepared for apoptosis and cell cycle analysis.

For cell cycle analysis, 1 mL of pre-cooled 70% ethanol was added, and the cells were fixed overnight at −20 °C. Next, fixed cells were washed with PBS and incubated with a staining solution containing RNase A (50 μg/mL) and propidium iodide (PI) (50 μg/mL) in PBS for 30 min at room temperature. The cellular DNA content was analyzed with a FACSCalibur^®^ flow cytometer (BD Biosciences, San Jose, CA, USA). At least 10,000 cells were used for each analysis, and the distribution of cells in each phase of the cell cycle was displayed using histograms.

For apoptosis analysis, HCT116 cells were treated with the test compound for 48 h. After incubation, the cells were collected and washed twice with PBS. The cells were stained with Annexin V-FITC and propidium iodide (PI) solution using an Annexin V-FITC apoptosis detection kit (BD Biosciences) according to the manufacturer’s instructions. In brief, HCT116 cells were diluted to 1 × 10^6^ cells/mL. A 100 μL aliquot of cell suspension was transferred into a 15 mL round-bottom polystyrene tube, and 5 μL of PI solution were added to the cell suspension, which was further incubated for 20 min at room temperature in the dark. Stained cells were diluted with binding buffer and immediately analyzed by flow cytometry.

### 3.5. RNA Isolation and Real-Time Reverse Transcript-Polymerase Chain Reaction (RT-PCR)

RT-PCR was used to determine the gene expression of HIF-1α in HCT116 cells. Briefly, HCT116 cells (2 × 10^5^ cells/mL) were cultured in 36-mm dishes for 24 h. The cells were then treated with various indicated concentrations of SA for an additional 8 h, with or without CoCl_2_. Total cellular RNA was extracted with TRIzol reagent according to the manufacturer’s instructions. The total RNA (1 μg) that was isolated from the cells was used for reverse transcription reaction with Reverse Transcription Reagents. The cDNA was reverse transcribed at 42 °C for 60 min with 0.5 μg of oligo (dT)_15_ in a reaction volume of 20 μL using the reverse transcription system (Promega, MI, USA). Specific gene primers were designed and custom synthesized by Bioneer Corporation (Daejeon, Korea); HIF-1α forward: 5′-GATAGCAAGACTTTCCTCAGTCG-3′, reverse: 5′-TGGCCTCATATCCCATCAATTC-3′ and GUSβ forward: 5′-CTACATCGATGATGACATCACCGTCAC-3′, reverse: 5′-TGCCCTTGACAGAGATCTGGTAA-3′. Real-time PCR was conducted using a MiniOpticon system (Bio-Rad, Hercules, CA, USA); each PCR amplification included 5 μL of reverse transcription product, iQ SYBR Green Supermix (Bio-Rad, Hercules, CA, USA), and primers in a total volume of 20 μL. The following standard thermo cycler conditions were employed: 95 °C for 20 s prior to the first cycle; 40 cycles of 95 °C for 20 s, 56 °C for 20 s, and 72 °C for 30 s; 95 °C for 1 min; and 55 °C for 1 min. The threshold cycle (C_T_), indicating the fractional cycle number at which the amount of amplified target gene reached a fixed threshold for each well, was determined using the MJ Opticon Monitor software package (Bio-Rad, Hercules, CA, USA). Relative quantification, representing the change in gene expression in real-time quantitative PCR experiments between a sample-treated group and the untreated control group, was calculated by the comparative C_T_ method in accordance with previously described methods [32]. The data were analyzed by evaluating the expression 2^−ΔΔCT^, where ΔΔC_T_ = (C_T_ of target gene − C_T_ of housekeeping gene) treated group − (C_T_ of target gene − C_T_ of housekeeping gene) untreated control group. For the treated samples, the evaluation of 2^−ΔΔCT^ represents the fold change in gene expression relative to the untreated control, normalized to a housekeeping gene (GUSβ).

### 3.6. Western Blot Analysis

HCT116 cells (2 × 10^5^ cells/mL) were placed in a 36-mm culture dish and incubated for 24 h. A variety of concentrations of SA were then added, with or without CoCl_2_, and the cells were cultured for the indicated time points before being digested. The protein was extracted with lysis buffer, and the protein concentrations were determined using the bicinchoninic acid (BCA) method. A 40 μg protein sample was collected from each group, boiled for 10 min, loaded onto 10% SDS-PAGE gels, and then transferred to PVDF membranes with electroblotting. Membranes were blocked for 1 h with 5% fat-free milk at room temperature, rinsed with PBS, and incubated with diluted primary antibodies 1:1000 or 1:500 overnight at 4 °C. Then, the membranes were incubated with specific secondary antibodies (1:1000) for 2 h and rinsed with PBS. The expression of β-actin was used as an internal standard. Proteins were detected with an enhanced chemiluminescence detection kit from GE Healthcare (Little Chalfont, UK) and an LAS-4000 Imager (Fuji Film Corp., Tokyo, Japan).

### 3.7. Statistical Analysis

The data are presented as the mean ± SD for the indicated number of independently performed experiments. Statistical significance (*p* < 0.05) was assessed by one way analysis of variance (ANOVA) coupled with Dunnett’s *t*-test.

## 4. Conclusions

**Figure 6:**
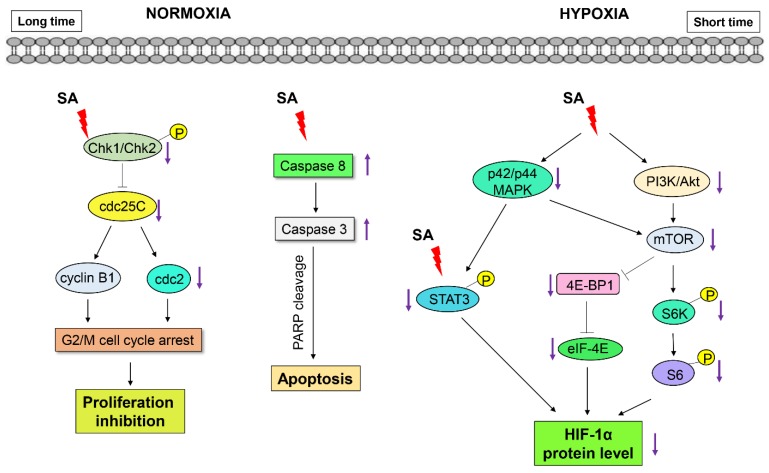
Proposed signaling pathways underlying the effects of SA on the suppression of HIF-1α and the induction of cell death in human colorectal cancer cells.

Salternamide A was identified for the first time as an inhibitor of HIF-1α accumulation under hypoxic conditions in cancer cells (Figure 6). The translational regulation of HIF-1α accumulation by SA was partly associated with the down-regulation of the axis of the PI3K/mTOR/STAT3 signaling pathways. The anti-proliferative activity of SA in HCT116 colorectal cancer cells was also associated with G2/M cell cycle arrest and apoptotic/autophagic cell deaths. Therefore, SA is a leading candidate for the development of anticancer agents, and these mechanisms will be a key therapeutic target for pharmacologic and therapeutic intervention in HIF-1α-driven tumor growth and cell death.

## Abbreviations

HIF-1: hypoxia inducible factor-1; MG132: Z-Leu-Leu-Leu-al; Hsp90: heat-shock protein 90; VHL: Von Hippel-Lindau; Akt: oncogene from AKR mouse thymoma; PI3K: phosphoinositide 3-kinase; mTOR: mammalian target of rapamycin; p70S6K: p70 S6 kinase; 4E-BP1: initiation factor 4E-binding protein 1; RPS6: ribosomal protein S6; eIF4E: eukaryotic translation initiation factor 4E; VEGF: vascular endothelial growth factor; STAT3: signal transducer and activator of transcription-3; MAPK: mitogen-activated protein kinase; ERK: extracellular-signal-related kinase; LC3: light chain 3.
